# Myeloid/Lymphoid Neoplasm With FGFR1 Rearrangement Accompanying *RUNX1* and *NOTCH1* Gene Mutations

**DOI:** 10.3389/fonc.2019.01304

**Published:** 2019-11-22

**Authors:** Xiaoxue Wang, Xinyue Huang, Hui Pang, Sheng Xiao, Hongcang Gu, Heyang Zhang, Baixun Wang, Lijun Zhang, Xiaojing Yan

**Affiliations:** ^1^Department of Hematology, The First Hospital of China Medical University, Shenyang, Liaoning, China; ^2^Department of Pediatrics, University of Oklahoma Health Sciences Center, Oklahoma City, OK, United States; ^3^Department of Pathology, Brigham and Women's Hospital, Harvard Medical School, Boston, MA, United States; ^4^Broad Institute of MIT and Harvard, Cambridge, MA, United States

**Keywords:** FGFR1, MPN, MPAL, *Runx1*, Notch

## Abstract

**Background:** Myeloid/Lymphoid Neoplasm with FGFR1 Rearrangement is a rare kind of hematological malignant disease.

**Case presentation:** A 37-year-old male patient experienced three distinct disease stages from myeloproliferative neoplasm (MPN), T-cell lymphoblastic lymphoma (T-LBL) to a much more complexed phage of a mixed phenotype acute leukemia (MPAL). Both genetic and genomic alternations were detected including chromosomal abnormality and genic mutations.

**Result:** Karyotyping and fluorescence *in situ* hybridization (FISH) analysis of either bone marrow or lymph node sample confirmed the presence of the *FGFR1* rearrangement. Amplifications of *RUNX1, ERG*, and *U2AF1* genes were identified by next generation sequencing. Furthermore, a frame-shift variant of F330fs^*^>149 in the *RUNX1* gene and a missense mutation of R2263Q in *NOTCH1* were also detected.

**Conclusion:** The *FGFR1* rearrangement functions as a trigging oncogenic event. Then other genetic events such as *RUNX1* and/or *NOTCH1* alternations further lead to progression of disease with trilineage blasts assignment.

## Background

Myeloid/lymphoid neoplasms with fibroblast growth factor receptor-1 (*FGFR1*) rearrangement has been presented as an independent disease classification in the 2016 World Health Organization classification of tumors of hematopoietic and lymphoid tissues (1). Rearrangement of the *FGFR1* gene locus leads to more than 10 fused genes, which code for fusion proteins containing the N-terminal derived from a partner gene and the C-terminal of tyrosine kinase domain from the *FGFR1* gene, which causes constitutive activation of *FGFR1* (2). The patients with *FGFR1* rearrangement usually initiate with myeloproliferative neoplasm (MPN) and undergo through diverse clinical courses such as lymphoblastic lymphoma (LBL), acute lymphoblastic leukemia (ALL) or acute myeloid leukemia (AML) (3). Here, we reported an interesting and very rare case which presented sequentially as MPN, T-LBL in lymph nodes and mixed phenotype acute leukemia

(MPAL) in bone marrow. To the best of our knowledge, our case represents the first report of this disease with three distinct developmental phases.

## Case Presentation

**Figure 1:**
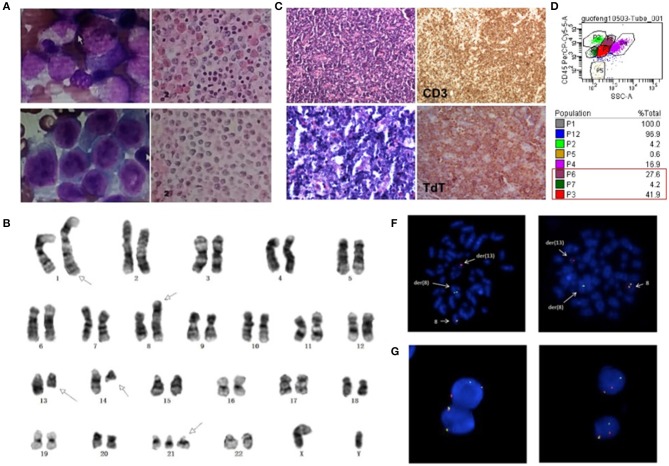
**(A)** Morphologic changes of BM smear and biopsy showed trilineage hypercellularity at initial onset. **(B)** Cervical lymph node biopsy with infiltration of T lymphoblasts. **(C)** BM smear showed markedly increased abnormal blasts and BM biopsy showed diffuse hyperplasia of immature cells at leukemic stage. **(D)** Flow cytometry analysis showed three separate clonal populations in BM sample. **(E)** G-banding karyotype of the bone marrow (BM) cells demonstrated the t(8;13)(p11.2;ql2.l); t(l;l4)(q42;q12) and trisomy 21. Arrows show the abnormal chromosomes. FISH analysis of BM cells **(F)** and lymph nodes **(G)** with an FGFR1 break-apart DNA probe revealing FGFR1 rearrangement (the 5'FGFR1 is labeled green, the 3'FGFR1 is labeled red).

A 37-year-old male patient was admitted to our center in August 2017 due to abnormal complete blood count (CBC) with WBC 34.1^*^10^9^/L and a hemoglobin (HGB) level of 205 g/L. Further bone marrow (BM) evaluation showed hypercellularity with trilineage growth including prominent granulocytic, erythroid and megakaryocytic proliferation (Figure 1A). Myelofibrosis was absent, also *JAK2 V617F/JAK2 exon 12/MPL/CALR* gene mutations or *BCR/ABL* fusion gene were not identified. The EPO level decreased to 0.82 mIU/ml, thus the clinical diagnosis of polycythemia vera (PV) was made by combining with BM biopsy findings and increased hemoglobin. After treatment of phlebotomy and hydroxyurea, patient's CBC dropped back normal.

Three months later, the patient came back to the hospital for bilateral cervical lymphadenopathy with fever and fatigue. Ultrasonographic findings suggest splenomegaly and generalized lymphadenopathy on both sides of the diaphragm. Immunohistochemical staining for cervical lymph node biopsy displayed as diffused abnormal proliferative lymphoblastic cells with CD3(+) TdT(+) CD99(+) CD4(+) Ki67(70%), while few scattered cells were positive for MPO, CD117 and CD8 (Figure 1B). Then, the patient was diagnosed as T-lymphoblastic lymphoma (LBL). The CBC of the patient showed a WBC count of 27.16^*^10^9^/L and a HGB level of 200 g/L. Intriguingly, bone marrow aspirate revealed hypercellularity with predominant blasts (Figure 1C) and flow cytometry showed three separate clonal populations (Figure 1D): (1) The majority of blasts were cells with ambiguous lineage (P3 group, 41.9%), mainly expressed CD33, CD34, CD13, HLA-DR, CD123; partially expressed CD117, CD22, CD19, CD10, cCD79a, and MPO. (2) The myeloid subset (P6 group, 27.6%) mainly expressed CD33, CD13, HLA-DR, CD123, CD4, CD11c and partially expressed CD14 and CD22. (3) The B-lymphoblast subset (P7 group, 4.2%) mainly expressed CD34, CD19, CD22, CD10, cCD79a, HLA-DR and partially expressed CD33 and CD13. Accordingly, the patient was diagnosed as MPAL. Karyotyping analysis of BM sample illustrated as 47, XY, t(1;14)(q42;q12), t(8;13)(p11.2;q12), +21 [20] (Figure 1E) and fluorescence *in situ* hybridization (FISH) analysis using the *FGFR1* break-apart probe confirmed the presence of the *FGFR1* rearrangement in all metaphase cells scored (Figure 1F). Then, next generation sequencing (NGS) assay of 118 commonly mutated genes in hematological disorders (listed in Supplementary Table 1) identified the amplifications of *RUNX1, ERG*, and *U2AF1* genes on chromosome 21p. Remarkably, a frame-shift variant of F330fs^*^>149 in the *RUNX1* gene and a missense mutation of R2263Q in *NOTCH1* were also detected. However, the NGS assay didn't identify any gene mutation using the BM samples collected during the patient's first visit. Interestingly, *FGFR1* rearrangement was also detected in 36% (18/50) of cells in lymph node biopsy sample (Figure 1G). Based on the clinical course and laboratory findings, the patient was finally diagnosed as myeloid/lymphoid neoplasm with *FGFR1* rearrangement.

The patient was treated with CHOP and hyper-CVAD chemotherapy. However, complete remission (CR) was not achieved. The patient could not find suitable HLA-matched donor for allogeneic hematopoietic stem cell transplantation (HSCT), and no further treatment were performed. Unfortunately, the patient died 10 months after onset.

## Discussion

**Figure 2:**
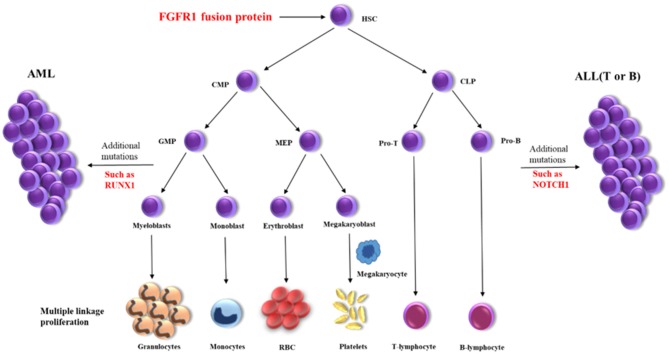
Schematic diagram of the disease progression mechanism. HSC, hematopoietic stem cells; CMP, conu11on myeloid progenitor; CLP, common lymphoid progenitor; GMP, granulocyte macrophage progenitor; MEP, megakaryocyte erythroid progenitor; Pro-T, T progenitor cells; Pro-B, B progenitor cells.

Myeloid/lymphoid neoplasms with *FGFR1* rearrangement, also known as the 8p11 myeloproliferative syndrome (EMS) or stem cell leukemia/lymphoma syndrome (SCLL), is extremely rare with around 100 cases reported until now which represented as MPN or more complicated situations (4, 5). It has been reported that FGFR1 could activate multiple signaling pathways as a receptor tyrosine kinase and constitutive activation of FGFR1 by gene fusion is a critical event in oncogenesis (2, 4). The prognosis of patients were generally poor and HSCT is probably the best choice for a long-term survival (5, 6). The presence of multiple lineage disorders suggests the involvement of stem cells for patients harboring *FGFR1* rearrangement (3). Gain of an additional copy of chromosome 21 is associated with progression of EMS, but its exact role remains unclear (2). Here we detected the amplifications of *RUNX1, ERG*, and *U2AF1* genes on chromosome 21p, which have been reported playing important roles in the pathogenesis of hematological malignancies (7, 8). A high frequency of *RUNX1* mutation can be seen in patients with *FGFR1* rearrangement (9), which have also been detected in a variety of hematological malignancies. Here, we also identified a missense variant of *NOTCH1* and this gene is recurrently found mutated in T-ALL but firstly reported in EMS. While, *RUNX1* and *NOTCH1* mutations were identified in the late stage of the disease (MPAL), but not in the initial stage of the disease (MPN). These findings indicated that FGFR1 rearrangement might be the initial oncogenic event in the hematopoietic pluripotent stem cells, which promote the trilineage hematopoiesis. Then other genetic events such as *RUNX1* and/or *NOTCH1* abnormalities further accelerated the progress of the disease, resulting in acute leukemia with trilineage blasts assignment (Figure 2). The abnormal hematopoietic stem cells present various phenotypes because of distinct microenvironments, such as lymphoma in lymph nodes or leukemia/MPN in bone marrow.

## Conclusion

Our case provides a typical clinico-pathogenic model of myeloid/lymphoid neoplasm with *FGFR1* rearrangement, which has not been reported previously. More clinical data are needed to identify the pathogenesis and interaction mechanism as well as optimal therapeutic approach to improve clinical outcomes.

## Data Availability Statement

All datasets generated for this study are included in the article/Supplementary Material.

## Ethics Statement

The studies involving human participants were reviewed and approved by China Medical University. The patients/participants provided their written informed consent to participate in this study.

## Consent

Written informed consent was obtained from the patient for publication of this case report and the accompanying images.

## Author Contributions

XW collected and analyzed all data, wrote the draft. HP and HG responsible for the revision. XH, HZ, BW, and LZ cared for the patient and provided clinical history. SX performed the Karyotype, FISH, and NGS. XY designed the study and responsible for the revisions.

### Conflict of Interest

The authors declare that the research was conducted in the absence of any commercial or financial relationships that could be construed as a potential conflict of interest.

## References

[1] Arber DA Orazi A Hasserjian R Thiele J Borowitz MJ Le Beau MM. The 2016 revision to the World Health Organization classification of myeloid neoplasms and acute leukemia. Blood. (2016) 127:2391–405. 10.1182/blood-2016-03-643544. 27069254

[2] Jackson CC Medeiros LJ Miranda RN. 8p11 myeloproliferative syndrome: a review. Hum Pathol. (2010). 41:461–76. 10.1016/j.humpath.2009.11.003 20226962

[3] Kumar KR Chen W Koduru PR Luu HS. Myeloid and lymphoid neoplasm with abnormalities of FGFR1 presenting with trilineage blasts and *RUNX1* rearrangement: a case report and review of literature. Am J Clin Pathol. (2015) 143:738–48. 10.1309/AJCPUD6W1JLQQMNA 25873510

[4] Macdonald D Reiter A Cross NC. The 8p11 myeloproliferative syndrome: a distinct clinical entity caused by constitutive activation of FGFR1. Acta Haematol. (2002) 107:101–7. 10.1159/000046639 11919391

[5] Strati P Tang G Duose DY Mallampati S Luthra R Patel KP, Myeloid/lymphoid neoplasms with FGFR1 rearrangement. Leuk Lymphoma. (2018) 59:1672–6. 10.1080/10428194.2017.1397663 29119847

[6] Umino K Fujiwara SI Ikeda T Toda Y Ito S Mashima K. Clinical outcomes of myeloid/lymphoid neoplasms with fibroblast growth factor receptor-1 (FGFR1) rearrangement. Hematology. (2018) 23:470–7. 10.1080/10245332.2018.1446279 29486661

[7] Ilagan JO Ramakrishnan A Hayes B Murphy ME Zebari AS Bradley P. U2AF1 mutations alter splice site recognition in hematological malignancies. Genome Res. (2015) 25:4–26. 10.1101/gr.181016.114 25267526 PMC4317169

[8] Mandoli A Singh AA Prange KHM Tijchon E Oerlemans M Dirks R. The hematopoietic transcription factors *RUNX1* and ERG prevent AML1-ETO oncogene overexpression and onset of the apoptosis program in t(8;21) AMLs. Cell Rep. (2016) 17:2087–100. 10.1016/j.celrep.2016.08.082 27851970

[9] Baer C Muehlbacher V Kern W Haferlach C Haferlach T Molecular genetic characterization of myeloid/lymphoid neoplasms associated with eosinophilia and rearrangement of PDGFRA, PDGFRB, FGFR1 or PCM1-JAK2. Haematologica. (2018) 103:e348–50. 10.3324/haematol.2017.187302 29567772 PMC6068021

