# Supplementary material for: Myeloid/Lymphoid Neoplasm With FGFR1 Rearrangement Accompanying RUNX1 and NOTCH1 Gene Mutations
Source: Front Oncol. 2019 Nov 22;9:1304. doi: 10.3389/fonc.2019.01304 (PMC6883488; doi:10.3389/fonc.2019.01304)

Supplementary table 1: Next generation sequencing (NGS) assay of 118 commonly mutated genes in hematological disorders


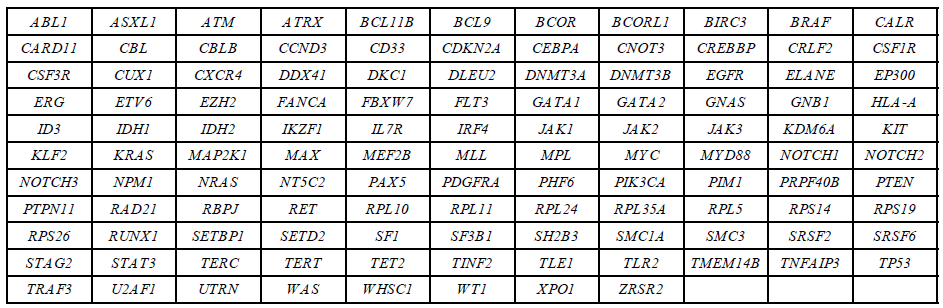

Supplement: Supplementary file 1 [file Table_1.DOCX]
